# Divergent Photosynthetic Strategies of *Lupinus polyphyllus* and *Helleborus viridis* During Cold Acclimation and Freezing–Thaw Recovery

**DOI:** 10.3390/plants14040607

**Published:** 2025-02-17

**Authors:** Pengyuan Xie, Yining Zhao, Xin Zhao, Linbo Xu, Kai Wang, Ruidong Jia, Yaping Kou, Hong Ge, Wenjun Wang, Shuhua Yang

**Affiliations:** 1 State Key Laboratory of Vegetable Biobreeding, Key Laboratory of Biology and Genetic Improvement of Flower Crops (North China), Ministry of Agriculture and Rural Affairs, Institute of Vegetables and Flowers, Chinese Academy of Agricultural Sciences, Beijing 100081, China; xiepengyuan@haas.cn (P.X.); zyning10@126.com (Y.Z.); zhaoxin01@caas.cn (X.Z.); jiaruidong@caas.cn (R.J.); kouyaping@caas.cn (Y.K.); gehong@caas.cn (H.G.); 2 Institute of Industrial Crops, Heilongjiang Academy of Agricultural Sciences, Harbin 150086, China; 3 Northern Agriculture and Livestock Husbandry Technology Innovation Center, Institute of Grassland Research, Chinese Academy of Agricultural Sciences, Hohhot 010010, China; xulinbo@caas.cn (L.X.); wangkai01@caas.cn (K.W.)

**Keywords:** photosynthesis, photoinhibition, antioxidant system, cold tolerance

## Abstract

Low temperatures can significantly affect the growth of ornamental plants, emphasizing the importance of improving their cold tolerance. However, comparative studies on the photosynthetic responses of sun and shade plants to low temperatures remain limited. In this study, gas exchange, chlorophyll fluorescence in Photosystem II (PSII) and Photosystem I (PSI), the antioxidant system, the osmoregulator substance, and lipid peroxidation were investigated in the shade plant *Helleborus viridis* (*Hv*) and the sun plant *Lupinus polyphyllus* (*Lp*) during cold acclimation (CA) and the freezing–thaw recovery (FTR). The CA treatment significantly declined the net photosynthetic rate (Pn) and the maximum photochemical efficiency of PSII (Fv/Fm) in *Hv* and *Lp*, indicating the photoinhibition occurred in both species. However, *Hv* exhibited a much better photosynthetic stability to maintain Pn, Fv/Fm, and carboxylation efficiency (CE) than *Lp* during CA, suggesting that *Hv* had a greater photosynthetic resilience compared to *Lp*. Furthermore, *Hv* preferred to maintain Pn, Fv/Fm, the actual photosynthetic efficiency of PSII (Y(II)), and the actual photosynthetic efficiency of PSI (Y(I)) to consistently provide the necessary energy for the carbon assimilation process, while *Lp* tended to divert and dissipate excess energy by thermal dissipation and cyclic electron flow during CA. Moreover, there were higher soluble sugar contents in *Hv* in comparison to *Lp*. These traits allowed *Hv* to recover photosynthetic efficiency and maintain cellular integrity better than *Lp* after the freezing stress. In conclusion, CA significantly reduced the photosynthetic capacity and led to the divergent photosynthetic strategies of both species, which finally resulted in a different freezing tolerance after the freezing–thaw recovery. These findings provide insights into the divergent photoprotective strategies of sun and shade plants in response to cold temperatures.

## 1. Introduction

Low temperatures, including chilling (0–15 °C) and freezing (<0 °C), are significant abiotic stresses that negatively affect the growth and development, limit the geographical distribution of plant species, and decrease crop yields worldwide [1,2]. Many plant species have developed the ability to enhance cold hardiness through cold acclimation (CA), a complex adaptive process initiated by an exposure to non-freezing temperatures [3]. CA involves the activation of pathways such as calcium (Ca^2+^) signaling, mitogen-activated protein kinase (MAPK) cascades, and the C-repeat binding factor-cold regulated (CBF-COR) signaling pathway, leading to physiological and biochemical adjustments [4,5]. These adjustments include stabilizing the photosynthetic apparatus, mitigating oxidative damage through reactive oxygen species (ROS)-scavenging systems, and accumulating osmoregulatory substances such as soluble sugars and proline to maintain cellular homeostasis and prevent ice crystal formation [6,7].

Photosynthesis, the process by which plants capture light energy to form carbohydrates, is highly susceptible to cold stress [8]. Low temperatures may impair photosynthesis by affecting stomatal function, electron transport, and carbon assimilation. In particular, the inhibition of photosystem II (PSII) disrupts the balance between energy capture and utilization, leading to a reduced photosynthetic efficiency [9]. In tobacco, low temperatures reduced the maximum quantum yield (Fv/Fm), PSII efficiency, and cyclic electron flow (CEF), and an increase in non-photochemical quenching (NPQ) helped to dissipate the excess energy and mitigate photoinhibition [10]. However, many evidences suggest that photosystem I (PSI) is more sensitive to low temperatures than PSII [11,12]. For instance, Ivanov et al. found that PSI photochemistry is less inhibited than PSII photochemistry in *Pinus sylvestris* L., enhancing the cyclic electron transfer around PSI that can provide electrons to synthesize ATP under conditions where the rates of photosynthetic linear electron transport are limited at the level of PSII [11]. In addition, photorespiration and the Mehler reaction are two other protective mechanisms in plants for avoiding photoinhibition. The Miller reaction can reduce oxygen to superoxide anion(O_2_^−^) by the excitation energy of PSII, a process that consumes excess electrons and reduces the damage to PSI [13]. Subsequently, superoxide dismutase (SOD) can catalyze O_2_^−^ into hydrogen peroxide (H_2_O_2_) and the latter is removed efficiently by catalase (CAT) and guaiacol peroxidase (POD), and the ascorbate–glutathione cycle involved in the antioxidant enzymes of ascorbate peroxidase (APX) and glutathione reductase (GR) synergistically maintain the integrity of photosynthetic apparatus [14].

CA can mitigate the photodamage under cold stress, which in turn promotes recovery. For instance, the CA treatment improved the cold resistance in tobacco leaves by preserving Fv/Fm, reducing PSII inhibition, and protecting receptor-side electron transport [10]. CA also stimulated ROS accumulation, boosted antioxidant defenses, increased osmotic substance levels, and improved the efficiency of cyclic electron flow (CEF), while effectively managing energy dissipation through the regulation of the quantum yield of regulated non-photochemical energy loss in PSII (Y(NPQ)). Additionally, CA significantly alleviated the inhibition of photosynthesis, enhanced the antioxidant system, and improved the adaptation and resistance of citrus seedlings to cold stress [15].

Plants exhibit different photosynthetic adaptations shaped by their light environments [16]. Sun plants tend to have the greater potential for photosynthetic electron transport compared with shade plants, which are more adept at capturing and optimizing light [17]. Sun plants, such as *Verbascum thapsus* and *Thymus vulgaris*, are adapted to high-light intensities, exhibiting a higher light saturation point (LSP) and light compensation point (LCP) [18]. However, *Athyrium pachyphlebium*, with a considerable shade tolerance, exhibited a lower light-saturated photosynthetic rate (P max), LCP, and LSP, with a higher electron transport rate (ETR) and chlorophyll b (Chl b) contents, indicating a greater efficiency in absorbing and utilizing light energy in low light [19]. A survey of photosynthetic pigments, including 86 species from 64 families, found that shade plants could accumulate more neoxanthin to capture light energy and resist photodamage than sun plants [20]. Low temperatures may significantly modify the photosynthetic performances in different plants. Although there were many evidences on the photosynthetic differences among the heliophilous and shade-tolerant species, the comparative analysis on cold-temperature-induced photosynthetic divergence in sun and shade plants remains limited. It is very necessary to compare the low-temperature-affected photosynthetic strategies in sun and shade plants.

*Lupinus polyphyllus*, belonging to the Leguminosae family, are of excellent landscape value due to their pretty inflorescence and vibrant flower colors. *Helleborus viridis*, belonging to the Ranunculaceae family, is becoming a new fashionable commercial flower with a notable flower type and cold resistance. In this study, the sun plant *Lupinus polyphyllus* and the shade plant *Helleborus viridis* were selected as materials to enrich a comparative study on cold-induced photosynthetic responses between sun and shade plants. Physiological changes in gas exchange, chlorophyll fluorescence, ROS-scavenging enzyme activity, and osmoregulatory compound accumulation were assessed in the leaves of *Lupinus polyphyllus* and *Helleborus viridis* during 14 d of CA and 1 d of freezing–thaw recovery (FTR) after CA. This study aims to elucidate how CA and the subsequent FTR influence photosynthetic performance and photoprotective strategies in these two perennial ornamental species. The findings will contribute to a comprehensive understanding of the divergent photosynthetic mechanisms underlying sun and shade plants in response to cold temperatures, which may further provide a theoretical foundation for the cold tolerance mechanism of perennial ornamental plants.

## 2. Results

### 2.1. Light and CO_2_ Response Curves

To understand the differences in light and CO_2_ utilization between the sun plant *Lp* and the shade plant *Hv* during cold acclimation, we measured and compared their light and CO_2_ response parameters. After 14 days of cold acclimation, both species exhibited changes in their light and CO_2_ response parameters. As shown in Table 1, the apparent quantum yields (AQE) and dark respiration rate (Rd) remained similar between species at 23/16 °C. However, the light saturation point (LSP) and light compensation point (LCP) were significantly higher in *Lp* compared to *Hv* under the same conditions. Cold acclimation led to significant declines in the AQE and Rd in both species, while the LSP and LCP followed similar trends. There were no significant differences in the AQE, Rd, and LCP between CA-*Lp* and CA-*Hv*, but the LSP was notably higher in CA-*Lp*.

The main parameters of the CO_2_ response curves are listed in Table 1. The photorespiration rate (Rp) showed no difference between the two species under the same temperature conditions. However, the values of carboxylation efficiency (CE), the CO_2_ saturation point (CSP), and the CO_2_ compensation point (CCP) were significantly higher in NA-*Lp* compared to NA-*Hv* at 23/16 °C. Cold acclimation significantly reduced the CE in both species but did not alter the CSP. The CCP increased significantly in both CA-*Lp* and CA-*Hv*, with no significant differences between them.

### 2.2. Leaf Gas Exchange

**Figure 1:**
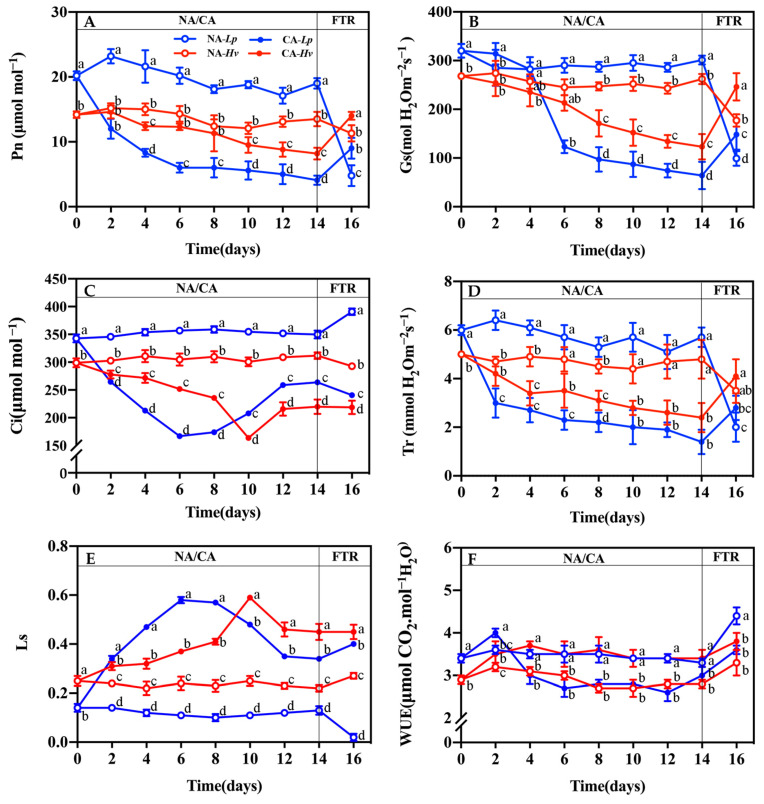
Changes in the leaf (**A**) net photosynthetic rate (Pn), (**B**) stomatal conductance (Gs), (**C**) intercellular CO_2_ concentration (Ci), (**D**) transpiration rate (Tr), (**E**) stomatal limitation (Ls) and (**F**) water use efficiency (WUE) in the seedlings of *Lupinus polyphyllus* and *Helleborus viridis* during cold acclimation (CA) and the following freezing–thaw recovery (FTR). Values are the means of five biological replicates ± SE (*n* = 5). Means followed by the different letters indicate the significant differences among the treatments at *p* < 0.05.

Comparing the leaf gas exchange parameters of *Lp* and *Hv* provides insights into how the sun and shade plants respond to cold acclimation and freezing–thaw recovery. Leaf gas exchange parameters maintained steadily in both species after 14 days of growth at 23/16 °C (Figure 1). There was a significantly higher Pn in *Lp* than *Hv* with the NA treatment (Figure 1A). However, the Pn decreased in both species and reached the significant differences from the 2nd day in *Lp* and the 10th day in *Hv* during CA. As a result, the Pn values of CA-*Lp* were significantly lower from the 4th day than that of CA-*Hv*. After a 24 h freezing–thaw recovery (FTR), the Pn did not change significantly in NA-*Hv* but dramatically decreased in NA-*Lp*. CA-*Lp* and CA-*Hv* significantly increased in the Pn after the FTR treatment. Moreover, the Pn of CA-*Hv* fully recovered to the same levels as NA-*Hv* before the FTR treatment. This difference implies that the shade plant *Hv* was better able to maintain its photosynthetic rate under cold stress, highlighting a key difference in their gas exchange-based photosynthetic strategies.

Stomatal conductance (Gs) and the transpiration rate (Tr) showed similar trends in the Pn in both species and different treatments (Figure 1B,D). The leaves presented significantly higher Gs after the 6th day but a significantly higher Tr before the 6th day in NA-*Lp* than NA-*Hv*. Gs significantly decreased from the 6th day in *Lp* and the 8th day in *Hv* during CA (Figure 1B). However, significant declines of the Tr were also observed from the 2nd day between NA-*Lp* and CA-*Lp*, and the 8th day between NA-*Hv* and CA-*Hv* (Figure 1D). As a result, there was a significantly lower Gs from the 6th day in CA-*Lp* than CA-*Hv* but no notable differences of the Tr between CA-*Lp* and CA-*Hv*. After the FTR treatment, Gs and the Tr significantly decreased in both species of the NA treatment but increased in those of the CA treatment. Nevertheless, the values of Gs and the Tr in both species did not reach the significant difference between NA and CA treatments except that there was a significantly higher Gs in CA-*Hv* than NA-*Hv* after the FTR treatment.

The intercellular CO_2_ concentration (Ci) was significantly higher in NA-*Lp* than NA-*Hv* during 14 days of growth at 23/16 °C (Figure 1C). During CA, the Ci initially decreased in both species but increased on the 6th day in *Lp* and the 10th day in *Hv*. Consequently, the Ci was significantly lower in CA-*Lp* than CA-*Hv* from the 4th to the 8th day but higher from the 10th to the 14th day. After the FTR, the Ci remained unchanged in NA-*Hv*, CA-*Lp*, and CA-*Hv* but increased significantly in NA-*Lp*. There were significantly higher results in the stomatal limitation value (Ls) in NA-*Lp* than NA-*Hv* during 14 days of growth (Figure 1E). In CA-*Lp*, the Ls peaked on the 6th day, while in CA-*Hv*, it peaked on the 10th day. Subsequently, the Ls decreased in both species but remained significantly different between the NA and CA treatments. The FTR treatment did not significantly alter the Ls in *Hv* or CA-*Lp* but caused a significant decrease in NA-*Lp*. The leaf water use efficiency (WUE) was higher in NA-*Lp* than NA-*Hv* (Figure 1F). The CA treatment significantly altered the WUE in both species starting from the 3rd day. After the FTR, the WUE increased significantly in NA-*Lp*, NA-*Hv*, and CA-*Lp*, while it remained unchanged in CA-*Hv*.

### 2.3. Leaf Chlorophyll Fluorescence in PSII

**Figure 2:**
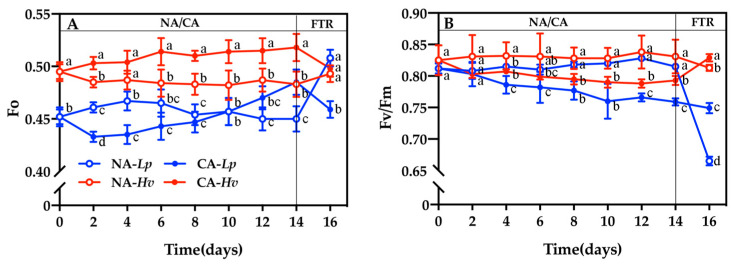
Changes in the (**A**) minimum fluorescence (Fo) and (**B**) maximum quantum yield of PSII (Fv/Fm) in the seedlings of *Lupinus polyphyllus* and *Helleborus viridis* during cold acclimation and the following freezing–thaw recovery. Values are the means of five biological replicates ± SE (*n* = 5). Means followed by the different letters indicate the significant differences among the treatments at *p* < 0.05.

**Figure 3:**
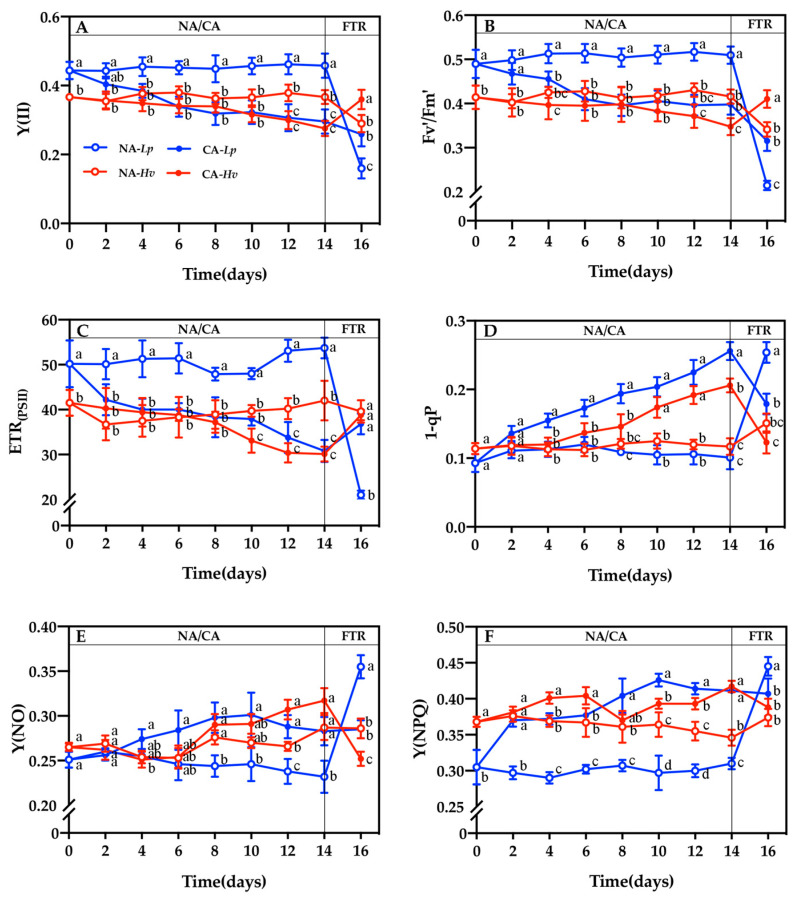
Changes in the (**A**) actual photosynthetic efficiency of PSII (Y(II)), (**B**) the efficiency of excitation energy capture by open PSII reaction centers (Fv′/Fm′), (**C**) PSII photosynthetic electron transport rate (ETR(PSII)), (**D**) PSII excitation pressure(1-qP), (**E**) the effective quantum yield of PSII (Y(NO)), and the (**F**) quantum yield of regulated non-photochemical energy loss in PSII (Y(NPQ)) in the seedlings of *Lupinus polyphyllus* and *Helleborus viridis* during cold acclimation and the following freezing–thaw recovery. Values are the means of five biological replicates ± SE (*n* = 5). Means followed by the different letters indicate the significant differences among the treatments at *p* < 0.05.

Analyzing the chlorophyll fluorescence parameters in PSII of *Lp* and *Hv* helps to elucidate the differences in their photosynthetic electron transport and photoprotective mechanisms in response to cold stress. Growth at 23/16 °C for 14 days did not affect the chlorophyll fluorescence parameters in PSII in either species (Figure 2 and Figure 3). The minimum fluorescence (Fo) was significantly lower in NA-*Lp* than NA-*Hv* throughout the 14-day period, except on the 6th and 10th days (Figure 3A). The Fo of CA-*Lp* decreased significantly on the 2nd day and then increased higher than that of NA-*Lp* on the 12th and 14th days. However, the Fo of CA-*Hv* increased significantly during CA except the 4th day in comparison to NA-*Hv*. As a result, a significantly lower Fo was observed in CA-*Lp* than CA-*Hv* during the whole CA. After the FTR treatment, the Fo increased sharply in NA-*Lp* while it decreased significantly in CA-*Lp*. Fo values in *Hv* exhibited similar trends as *Lp*, but did not significantly change in NA-*Hv* and CA-*Hv* compared with the values before the FTR treatment.

The maximum quantum yield of the PSII (Fv/Fm) values were around 0.808~0.838 and showed no difference between the two species under the NA treatment (Figure 3B). Fv/Fm decreased in both species during CA that presented the significantly lower values from the 4th day in CA-*Lp* than NA-*Lp*, and from the 8th day in CA-*Hv* than NA-*Hv*. Moreover, significantly lower Fv/Fm values were found in CA-*Lp* than CA-*Hv* after 12 days of cold acclimation. The FTR treatment did not alter Fv/Fm in NA-*Hv* and CA-*Lp*, but led to a sharply decline in CA-*Lp* and a significant increase in CA-*Hv*. Consequently, Fv/Fm showed significantly lower values in *Lupinus polyphyllus* than *Helleborus viridis* while significantly higher values in the CA treatment than NA treatment.

The actual photosynthetic efficiency of PSII (Y(II)), the efficiency of excitation energy capture by open PSII reaction centers (Fv′/Fm′), and the PSII photosynthetic electron transport rate (ETR(PSII)) were significantly higher in NA-*Lp* than in NA-*Hv* during the 14-day period at 23/16 °C (Figure 3A–C). These parameters declined in both species under CA, with significant differences in Y(II) and Fv′/Fm′ observed from the 4th day in CA-*Lp* and the 12th day in CA-*Hv*. ETR(PSII) declined significantly throughout CA in CA-*Lp* and from the 10th day in CA-*Hv*. Except for a higher Fv′/Fm′ on the 2nd day in CA-*Lp*, there were no significant differences in these parameters between CA-*Lp* and CA-*Hv*. After the FTR, Y(II) and Fv′/Fm′ decreased significantly in NA-treated plants but increased in CA-*Hv*, resulting in higher values in the CA-treated plants of both species. ETR(PSII) declined sharply in NA-*Lp* but increased significantly in CA-*Lp* and CA-*Hv*. A significantly lower ETR(PSII) was found in NA-*Lp* than NA-*Hv*, CA-*Lp* and CA-*Hv*.

The PSII excitation pressure (1-qP) did not differ between the two species under the NA treatment (Figure 3D). However, 1-qP increased notably from the 2nd day in CA-*Lp* and the 6th day in CA-*Hv*. As a result, significantly higher values of 1-qP were found on the 4th, 6th, 8th, and 14th day in CA-*Lp* than CA-*Hv*. After the FTR treatment, 1-qP significantly increased in NA-treated plants but declined in CA-treated plants, which led to a significantly higher 1-qP in NA-*Lp* than NA-*Hv*, CA-*Lp* and CA-*Hv*.

The leaves presented obviously lower in the effective quantum yield of the PSII (Y(NO)) values from the 8th day in NA-*Lp* than NA-*Hv* (Figure 3E). Furthermore, a significantly lower quantum yield of regulated non-photochemical energy loss in the PSII (Y(NPQ)) values was observed in NA-*Lp* than NA-*Hv* during 14 days of growth at 23/16 °C (Figure 3F). The CA treatment increased the values of Y(NO) and Y(NPQ) in both species. In *Lp*, significant differences were found in Y(NO) from the 6th day and Y(NPQ) from the 2nd day between the CA and NA treatments. In *Hv*, there were higher values of Y(NO) in CA-*Hv* than NA-*Hv* after 10 days of cold acclimation. Moreover, the CA treatment significantly increased Y(NPQ) values in CA-*Hv* than NA-*Hv* during CA except for the 2nd and 8th day. After the FTR treatment, Y(NO) and Y(NPQ) significantly increased in NA-*Lp*, whereas they did not change in CA-*Lp*. This resulted in significantly lower values of Y(NO) and Y(NPQ) in CA-*Lp* compared to NA-*Lp* after the FTR treatment. The FTR treatment did not affect Y(NO) and Y(NPQ) in NA-*Hv* but significantly decreased in CA-*Hv*. Finally, a significantly lower Y(NO) was observed in CA-*Hv* than NA-*Hv*.

### 2.4. Leaf Chlorophyll Fluorescence in PSI

**Figure 4:**
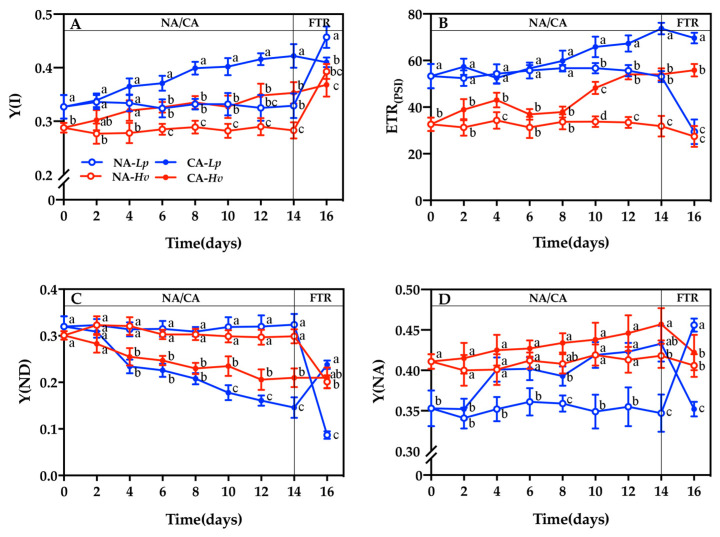
Changes in the (**A**) actual photosynthetic efficiency of PSI Y(I), (**B**) PSI photosynthetic electron transport rate (ETR(PSI)), (**C**) PSI non-photochemical energy dissipation due to the donor-side limitation (Y(ND)), and the (**D**) PSI non-photochemical energy dissipation due to the acceptor-side limitation (Y(NA)) in the seedlings of *Lupinus polyphyllus* and *Helleborus viridis* during cold acclimation and the following freezing–thaw recovery. Values are the means of five biological replicates ± SE (*n* = 5). Means followed by the different letters indicate the significant differences among the treatments at *p* < 0.05.

The NA treatment did not affect the PSI chlorophyll fluorescence parameters in either species (Figure 4). However, the actual photosynthetic efficiency of PSI Y(I) and the PSI photosynthetic electron transport rate (ETR(PSI)) were significantly higher in *Lp* than in *Hv* under the NA treatment (Figure 4A,B). During CA, Y(I) and ETR(PSI) increased in both species, with significant differences observed from the 6th day for Y(I) and the 10th day for ETR(PSI). Additionally, ETR(PSI) was significantly higher in CA-*Hv* than NA-*Hv* on the 4th day. After the FTR, Y(I) increased significantly in NA-treated seedlings but remained unchanged in CA-treated seedlings. Consequently, Y(I) was significantly lower in CA-*Lp* than NA-*Lp*. The FTR significantly decreased ETR(PSI) in NA-*Lp* but did not affect NA-*Hv*, CA-*Lp*, or CA-*Hv*, resulting in higher Y(I) and ETR(PSI) in CA-treated seedlings compared to NA-treated seedlings in both species.

The PSI non-photochemical energy dissipation due to the donor-side limitation (Y(ND)) did not exhibit the differences between the two species during 14 days of growth at 23/16 °C (Figure 4C). The CA treatment decreased Y(ND) and reached the significant lower values from the 4th day in both species. After the FTR treatment, Y(ND) significantly decreased in NA-*Lp* but increased in CA-*Lp*, which led to significantly higher Y(ND) in NA-*Lp* than CA-*Lp*. Y(ND) did not alter in CA-*Hv*, but significantly declined in NA-*Hv* and exhibited no difference between CA-*Hv* and NA-*Hv* after the FTR. The PSI non-photochemical energy dissipation due to the acceptor-side limitation (Y(NA)) was significantly lower in NA-*Lp* than NA-*Hv* (Figure 4D). During CA, Y(NA) increased in *Lp*, reaching significantly higher values from the 4th day. After the FTR treatment, Y(NA) significantly increased in NA-*Lp* but declined in CA-*Lp*, which resulted in a significantly lower Y(NA) in NA-*Lp* than CA-*Lp*. However, there were no differences of the Y(NA) between CA-*Hv* and NA-*Hv* during the NA/CA treatment or after the FTR treatment. The photosynthetic electron flow through ETR(I) in the leaves of *Lp* and *Hv* was significantly reduced during the CA treatment. CEF, indicated by the ratio of ETR(I)/ETR(II), increased sharply and the increment grew with time under CA.

### 2.5. Leaf H_2_O_2_ Contents and ROS-Scavenging Enzymes Activities

**Figure 5:**
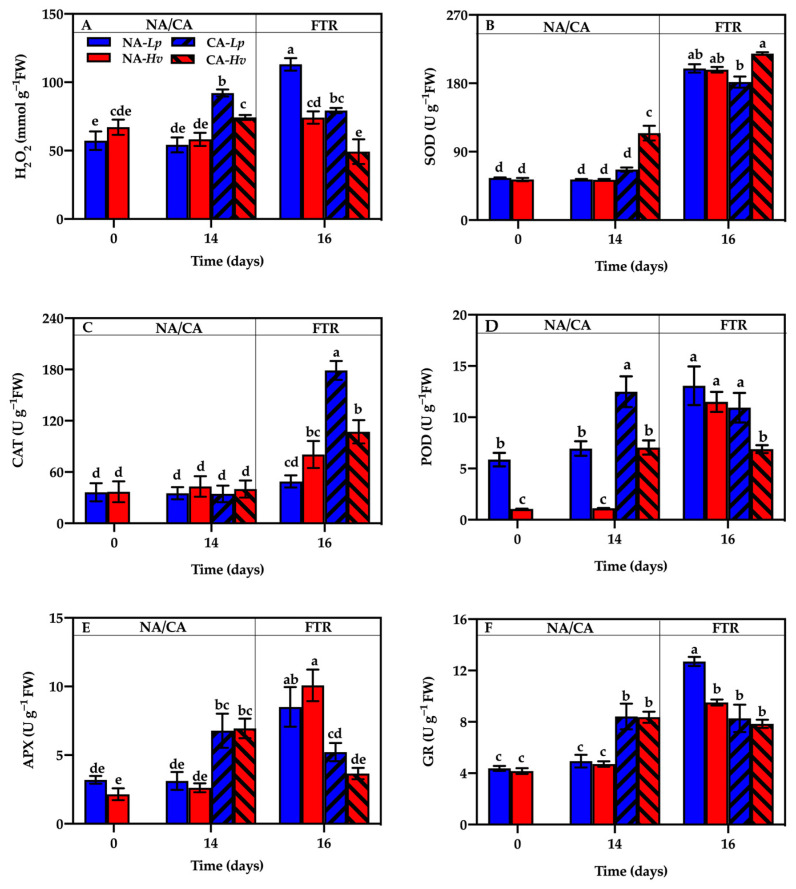
Changes in the (**A**) Hydrogen peroxide content (H_2_O_2_), the antioxidant enzyme activities of (**B**) superoxide dismutase (SOD), (**C**) peroxidase (POD), (**D**) catalase (CAT), (**E**) ascorbate peroxidase (APX), and (**F**) glutathione reductase (GR) in the seedlings of *Lupinus polyphyllus* and *Helleborus viridis* during cold acclimation and the following freezing–thaw recovery. Values are the means of five biological replicates ± SE (*n* = 5). Means followed by the different letters indicate the significant differences among the treatments at *p* < 0.05.

Comparing the hydrogen peroxide (H_2_O_2_) contents, ROS-scavenging enzyme activities, osmoregulatory substances, and injury indicators in *Lp* and *Hv* reveals how the sun and shade plants cope with oxidative stress and maintain cellular integrity during cold acclimation and recovery. The H_2_O_2_ contents were similar between the two species and remained stable after 14 days at 23/16 °C (Figure 5A). Cold acclimation significantly increased the H_2_O_2_ contents in both species, with higher contents in CA-*Lp* than CA-*Hv*. After the FTR, the H_2_O_2_ contents increased significantly in NA-*Lp* but decreased in CA-*Hv*, resulting in lower H_2_O_2_ contents in CA-treated seedlings compared to NA-treated seedlings in both species.

SOD activities did not alter in *Lp* but significantly increased in *Hv* after 14 days of CA (Figure 5B). The FTR significantly enhanced SOD activities in both NA- and CA-treated seedlings of both species, but no differences were observed between NA- and CA-treated seedlings within each species. There were higher POD activities in *Lp* than *Hv* during the NA treatment (Figure 5C). CA for 14 days significantly elevated POD activities in each species. After the FTR treatment, POD activities significantly increased in the NA treatment but did not alter in the CA treatment of each species. Finally, a significantly lower POD activity was observed in CA-*Hv* than NA-*Lp*, CA-*Lp*, and NA-*Hv*. There were not any effects of growth, temperature, and species on CAT activities (Figure 5D). The FTR significantly increased CAT activity in NA-*Hv*, CA-*Lp*, and CA-*Hv* but not in NA-*Lp*, resulting in a higher CAT activity in CA-*Lp* than NA-*Lp*.

APX and GR activities showed no species effects under the NA or CA treatment (Figure 5E,F). CA significantly increased APX and GR activities in both species. After the FTR, APX and GR activities increased significantly in NA-*Lp* and NA-*Hv*, while the APX activity decreased in CA-*Hv*. Consequently, higher APX and GR activities were observed in NA-treated seedlings compared to the CA-treated seedlings of *Lp*, with higher a APX activity in NA-*Hv* than CA-*Hv*.

### 2.6. Leaf Osmoregulatory Substances and Indicators of Injury

**Figure 6:**
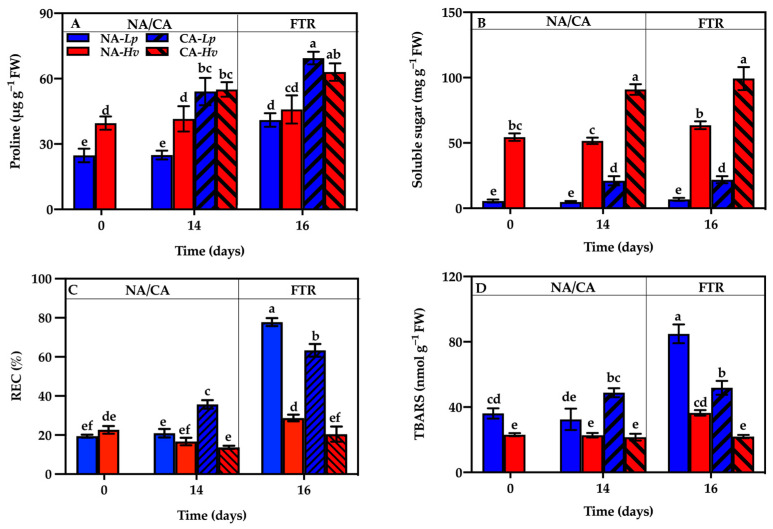
Changes in (**A**) proline, (**B**) soluble sugars, (**C**) relative electrical conductivity (REC), and (**D**) thiobarbituric acid reactive substances (TBARS) in the seedlings of *Lupinus polyphyllus* and *Helleborus viridis* during cold acclimation and the following freezing–thaw recovery. Values are the means of five biological replicates ± SE (*n* = 5). Means followed by the different letters indicate the significant differences among the treatments at *p* < 0.05.

There were significantly higher contents of proline and soluble sugars in *Hv* than in *Lp* during 14 days of growth at 23/16 °C (Figure 6A,B). CA significantly enhanced the contents of proline and soluble sugars in both species, which led to identical proline contents in the two species but maintained significantly higher contents of soluble sugars in CA-*Hv* than in CA-*Lp*. After the FTR, the proline content significantly increased in *Lp* but did not change in *Hv* (Figure 6A). Meanwhile, the content of soluble sugars remained unchanged in NA-*Lp*, CA-*Lp*, and CA-*Hv* but significantly increased in NA-*Hv* after the FTR treatment (Figure 6B). The CA-treated seedlings exhibited significantly higher contents of proline and soluble sugars than the NA-treated seedlings in both species.

There were similar relative electrical conductivity (REC) values between the two species and they remained steady under the NA treatment (Figure 6C). CA for 14 days significantly elevated the REC value in CA-*Lp* but did not change it in CA-*Hv*. After the FTR treatment, REC values dramatically increased in the NA- and CA-treated seedlings of *Lp* but only significantly increased in the NA-treated seedlings of *Hv*. As a result, significantly higher REC values were observed in the CA-treated seedlings than in the NA-treated seedlings of both species.

Thiobarbituric acid reactive substances (TBARS) contents showed a tendency very similar to the REC values (Figure 6D). There was no TBARS difference between the two species after 14 days of growth at 23/16 °C. The CA treatment significantly enhanced the TBARS content in CA-*Lp* but did not alter it in CA-*Hv*. After the FTR treatment, the TBARS contents significantly increased in the seedlings of *Lp* but remained unchanged in the seedlings of *Hv*. Consequently, there were higher TBARS contents in the CA-treated seedlings than in the NA-treated seedlings of both species.

## 3. Discussion

Low temperatures are one of the major challenges threatening plant survival, primarily due to their harmful effects on photosynthesis. CA can help mitigate chilling-induced damage and improve freezing tolerance in plants. In this study, we compared the changes in photosynthesis, osmoregulatory substances, and antioxidant enzyme activity between *Helleborus viridis* (*Hv*) and *Lupinus polyphyllus* (*Lp*), highlighting the different photosynthetic strategies employed by these two species under CA and FTR conditions.

Photosynthesis is essential for the growth, development, and metabolism of plants [21]. In this study, low temperatures significantly reduced the Pn in both *Hv* and *Lp*, with a more severe reduction observed in *Lp* than in *Hv* (Figure 1A). After the FTR, *Hv* restored its Pn to pre-stress levels, while *Lp* only recovered to about 50%, indicating that *Lp* is more sensitive to cold stress than *Hv*. During CA, the Ci initially decreased in both species but increased on the 6th day in *Lp* and the 10th day in *Hv* (Figure 1C), suggesting that there was a shift from stomatal to non-stomatal limitations in both species [22]. Stomatal closure limits CO_2_ assimilation, promotes an imbalance between photochemical activity at PSII and PSI, and subsequently decreases photosynthesis efficiency. *Lp* experienced stomatal limitation earlier than *Hv*, further confirming that *Lp* was more sensitive to cold stress than *Hv*. The LSP, LCP, CSP, and CCP in *Lp* were higher than those in *Hv* under NA conditions (Table 1), suggesting that *Lp* had a higher light requirement than *Hv* which was more adapted to medium or low light conditions. Under CA, the LCP and LSP showed more significant declines in *Lp* compared to *Hv*, highlighting its vulnerability to cold stress, which may explain the greater reduction in photosynthesis efficiency.

Low temperature may lead to photoinhibition, a decreased ability to excite energy capture, conversion capacity, and electron transfer activity in reaction centers, ultimately resulting in photodamage [10]. Fv/Fm is an excellent probe to indicate the degree of stress [23]. In this study, the Fv/Fm in both species decreased during the CA treatment, and the reduction of Fv/Fm values in *Lp* was more significant than *Hv* (Figure 2B), indicating that a chilling temperature caused PSII photoinhibition in both species and that the photoinhibition was more severe in *Lp*. Our results revealed that the Y(II) and ETR(PSII) values in both species at CA were lower than that in the control (Figure 3A,C), implying that the demagnesium chlorophyll (Pheo) on the donor side may have failed to promptly transfer electrons, preventing the primary acceptor of PSII (QA) from receiving them in time under chilling conditions [24]. Consequently, the accumulation of QA (as QA-) leads to the formation of P680+, which negatively impacts the function of PSII. The ETR(PSII) in *Lp* was significantly declined throughout the CA period and *Lp* performed more sensitively to chilling stress than from the 10th day in *Hv* (Figure 3C). Meanwhile, NPQ is one of the dominant pathways to dissipate excessive light energy including several quenching mechanisms [25]. During the CA treatment, the Y(NPQ) and Y(NO) of PSII significantly increased in both species (Figure 3E,F), implying a decrease of *Lp* and *Hv* leaf photosynthesis and a serious photodamage to PSII caused by chilling stress. However, *Lp* exhibited a higher NPQ than *Hv* to absorb the extra excitation energy in PSII, indicating that *Hv* used more energy for carbon fixation processes in photosynthesis instead of heat dissipation. This difference may be related to the photosynthetic adaptations of sun and shade plants. For instance, the sun plant *Aloe vera*, treated with high light, suffered less damage and a higher PSII photochemical efficiency under low temperatures compared with the plants under a low light condition [26]. However, the negative effects of low-temperature stress could be aggravated by high light as observed in the shade plant *Kobresia pygmaea* [27]. Our results found that CA enhanced PSII photochemistry in both species and improved their resilience of photosynthesis after freezing. Similarly, the frost tolerance of winter rye (*Secale cereale* cv. Musketeer) was associated with the adjustments in photosynthetic capacity, NPQ capacity and the tolerance to photoinhibition during CA, as well as the relative reduction state of photosystem II [28]. Fv/Fm, Y(II), ETR(PSII), Y(NO) and Y(NPQ) recovered to normal values only in CA-*Hv* after the FTR, reflecting that *Hv* could stabilize and restore PSII function better than *Lp* under low temperatures. In summary, *Lp* preferred to protect the photosynthetic apparatus through heat dissipation, while *Hv* tended to consume the light energy by improving PSII photochemistry and carbon assimilation.

Cold temperatures can also inhibit PSI photochemical activities with the reduced maximum quantum yield of electron transport through PSI, the pool of the photo-oxidizable reaction center pigment of PSI (P700), and the efficiency of P700 oxidation [29]. During CA, Y(I) and ETR(PSI) increased in both species (Figure 4A,B). The increases in Y(I) and ETR(I) could be related to the Mehler reaction, which channels excess electrons unavailable for NADP+ regeneration into oxygen reduction, thus protecting the photosynthetic machinery from becoming over-reduced [30]. Moreover, the CEF around PSI is another important strategy that generates additional ATP to protect PSI against photoinhibition even when PSII photochemistry efficiency is reduced [31,32,33]. A high ETR(I)/ETR(II) ratio of both species under low temperatures indicated that CA stimulated their CEF around PSI. The decreases of Y(ND) in *Lp* and *Hv* may be due to the impaired donor-side electron transport in PSI in plants (Figure 4C). Furthermore, the more serious decline of Y(ND) was observed in *Lp* than in *Hv* under CA (Figure 4D), reflecting that *Lp* presented the stronger CEF cycling around the PSI than *Hv*. Similar as CE, there was a greater enhancement of Y(NA) in *Lp* than *Hv* during CA, suggesting that *Lp* preferred to utilize the energy for CEF around PSI, whereas *Hv* preferred to consume the energy to reduce NADP^+^ to NADPH for CO_2_ fixation. As a result, *Hv* exhibited a greater PSI resilience and a more stable energy flow than *Lp* after the FTR.

Photoinhibition is mainly triggered by the ROS produced by excess light energy under stresses [34]. If the excessive ROS cannot be removed in time, the membrane lipid peroxidation will occur and finally lead to photodamage in plants [35]. In this experiment, CA significantly increased the H_2_O_2_ content in both species with *Lp*, showing a greater increase than *Hv* (Figure 5A), suggesting that *Lp* accumulated more ROS than *Hv*. The REC value and TBARS contents in *Lp* showed significant increases but no changes were observed in *Hv* over a 14-day CA period (Figure 6C,D), indicating *Lp* subsequently suffered a more severe photo-oxidative damage in leaves under low temperature than *Hv*. It is known that the antioxidant system is one of the important photoprotective mechanisms by removing excess ROS in plants [36]. In this study, the activities of POD, APX, and GR were significantly increased in both species (Figure 5D–F), but the activities of SOD were only significantly increased in Hv after 14 days of CA (Figure 5B), suggesting that both species tried to activate the antioxidant enzymes to scavenge ROS under low temperatures, and especially SOD might be the key enzyme in *Hv* for maintaining redox balance during CA. On the other hand, the osmoregulatory substances such as proline and soluble sugars can stabilize cellular structure, maintain the integrity of the photosynthetic membrane, and reduce freezing-induced dehydration [37,38]. In our results, CA significantly increased the content of proline and soluble sugars in both species (Figure 6A,B). Moreover, there was a much higher soluble sugar content in *Hv* than that in *Lp*, implying that the accumulation of soluble carbohydrates also might be the important mechanism to enhance cold tolerance in *Hv*.

## 4. Materials and Methods

### 4.1. Plant Materials and Treatments

The seedlings of *Lupinus polyphyllus* (*Lp*) and *Helleborus viridis* (*Hv*) were cultivated in the greenhouse of the Institute of Vegetables and Flowers, Chinese Academy of Agricultural Sciences. Two-year-old seedlings with a good growth and uniformity were selected and randomly assigned to two growth chambers. Each chamber housed 20 plants under controlled conditions of 380 μmol·mol^−1^ CO_2_ concentration (Ca), 600 μmol·m^−2^·s^−1^ photosynthetic photon flux density (PPFD), a 10/14 h photoperiod, and 23/16 °C (day/night) air temperature. After a three-day acclimation period, one chamber maintained these conditions as the non-acclimated (NA) treatment, while the day/night temperature in the other was lowered to 8/4 °C (day/night) for cold-acclimated (CA) treatment.

After 14 days of these treatments, seedlings from both chambers were subjected to a 24 h freezing treatment at −4 °C in a temperature cycling chamber (DRGL-400, Boyubaowei Scientific Instruments, Beijing, China). Following the freezing treatment, plants were returned to the NA growth chamber for a 24 h recovery period. The experiment followed a completely randomized block design with at least three biological replicates per treatment. To minimize micro-environmental effects, the pots were rotated every two days within each growth chamber.

### 4.2. Gas Exchange Measurements

Photosynthetic parameters were measured using the third fully expanded and mature leaf from each seedling. The Pn, Gs, Ci, Tr and water use efficiency (WUE = Pn/Tr) were measured using a CIRAS-3 Portable Photosynthesis System (PP Systems, Hitchin, UK) with a PLC3 Universal Leaf Cuvette (25mm × 7mm area) under a supplemental PAR of 800 μmol·m^−2^·s^−1^ at 10 am on each of the two days of the experiment [39]. The measurement carried out from 0 d to 14 d NA/CA treatments with every 2 d intervals, as well as the 16th day with the following 2 d freezing-recovery. Additionally, the stomatal limitation value (Ls) was calculated as follows: Ls = 1 − Ci/Ca [40].

### 4.3. Light and CO_2_ Response Curves Measurements

On the 14th day of the NA and CA treatments, photosynthesis–light response and CO_2_ response curves were measured. Light response curves were measured when CO_2_ concentrations were kept constant at 400 μmol·mol^−1^. During the light response measurements, the leaf was consecutively exposed to declined PPFD levels of 2500, 2000, 1800, 1600, 1400, 1200, 1000, 800, 600, 400, 200, 100, 50, and 0 μmol·m^−2^·s^−1^. Parameters including the AQE, LSP, LCP, and Rd were derived using a non-rectangular hyperbola equation [41].

The CO_2_ response curves were measured under a constant photosynthetically active radiation (PAR) of 1000 μmol·m^−2^·s^−1^ with stepwise changes in CO_2_ concentration (0, 50, 100, 200, 400, 600, 800, 1000, 1300, 1500, and 2000 μmol·m^−2^·s^−1^). The values of the CE, CSP, CCP and Rp were derived by fitting a hyperbolic correction model [42].

### 4.4. Chlorophyll Fluorescence Measurements

The chlorophyll fluorescence and P700 measurements were measured using the same leaves as for gas exchange. A DAUL-PAM-100 chlorophyll fluorometer (H. Walz, Effeltrich, Germany) in dual-channel mode was used. Before measurements, leaves were dark-adapted for 30 min. Fo was estimated under low modulated light (1 μmol·m^−2^·s^−1^). The intensity of the saturation pulse light (red light) and actinic light were set as 12,000 and 300 μmol quanta m^−2^·s^−1^, respectively. Fo and Fm were the minimum and maximum fluorescence yields of the dark-adjusted sample with all PSII centers open and closed, respectively. Fo′ and Fm′ were the minimum and maximum fluorescence yield of the illuminated sample with PSII centers open and closed, respectively. F is the fluorescence yields measured briefly before applying a saturation pulse.

Then, a saturating pulse of white light (300ms, 16000 μmol·m^−2^·s^−1^) was implemented to determine the maximum fluorescence at closed PSII centers in the dark-adapted state (Fm) and during actinic light illumination (Fm′). The minimum fluorescence in the light-adapted state (Fo′) was measured in the presence of far-red light after the actinic light was turned off. The P700 signals (P) may vary between a minimal (P700 fully reduced) and a maximal level (P700 fully oxidized). P700+ oxidation was monitored by absorbance changes in the near-infrared (830–875 nm). Pm indicated the maximal P700 signal observed upon full oxidation and was used to estimate the PSI activity. It was determined with the application of a saturation pulse (240 ms and 582 μmol photons m^−2^ s^−1^) after pre-illumination with far-red light. The Pm′, similarly to Pm, indicated the maximal P700 signal induced by the combined actinic illumination plus saturation pulse (240 ms and 582 μmol photons m^−2^ s^−1^).

Calculate according to the following formula [43]:

Fv/Fm = (Fm − Fo)/Fm

Y(II) = (Fm′ − F)/Fm′

Y(NPQ) = F/Fm′ − F/Fm

Y(NO) = F/Fm

Y(I) = (Pm′ − P)/Pm

Y(ND) = P/Pm

Y(NA) = (Pm − Pm′)/Pm.

### 4.5. Measurements of the H_2_O_2_ Contents and ROS-Scavenging Enzymes Activities

The H_2_O_2_ content was assayed spectrophotometrically at 405 nm by using an assay kit (A064-1-1, Nanjing Jiancheng, Nanjing, China) which based on the fact that H_2_O_2_ can interact with molybdic acid to form a complex compound [44]. Leaf H_2_O_2_ contents were determined using a H_2_O_2_ standard curve.

Antioxidant enzyme activities, including SOD, POD, and CAT, were measured using plant enzyme assay kits (Solarbio, Beijing, China) following the instructions [45]. Leaf samples (0.1 g) were homogenized in a 1 mL extraction buffer under frozen conditions. SOD activity was determined based on its inhibition of the xanthine oxidase reaction system, with one unit defined as 50% inhibition. POD activity was assessed by guaiacol oxidation in the presence of hydrogen peroxide, with absorbance measured at 470 nm. CAT activity was evaluated by monitoring the decrease in H_2_O_2_ absorbance at 240 nm, with one unit defined as the degradation of 1 μmol H_2_O_2_ min^−1^.

The APX and GR activity was evaluated according to Yang et al. [46], with modifications. The measurement was made using a plate spectrophotometer (Epoch2, BioTek, Winooski, VT, USA) in 96-well plates. Frozen leaflets were homogenized and centrifuged with 50 mM Tris-HCl (pH 7.0) containing 20% (*w*/*v*) glycerol, 1 mM reduced glutathione (GSH), and 5 mM MgCl_2_. The supernatant was prepared for the activity determination of APX and GR. The APX activity was measured spectrophotometrically by monitoring the decrease in absorbance at 290 nm as the ascorbate was oxidized. The reaction mixture for the GR activity contained 2.5 mM EDTA, 0.75 mM 5,5′-dithiobis (2-nitrobenzoic acid) (DTNB), 1 mM GSSG, 0.1 mM NADPH, and the enzyme extract in a 100 mM potassium phosphate buffer (pH 7.5). The decrease was recorded in absorbance at 412 nm. The enzyme activity of APX was calculated as l μmol AsA oxidized min^−1^ g^−1^ while the GR activity was expressed as l μmol NADPH catalyzed min^−1^ g^−1^.

### 4.6. Measurements of Proline and Soluble Sugars Contents

The proline content in leaves was determined by essentially following Bates L [47]. The measurement was made using a plate spectrophotometer (Epoch2, BioTek, Winooski, VT, USA) in 96-well plates at a wavelength of 520 nm. The leaves were homogenized with 0.1 g in 0.9 mL of sulfosalicylic acid (3%) and the homogeneous mixture was centrifuged at 3500 rpm for 10 min at 4 °C. The extract was transformed to a new tube and mixed with 200 mL of acid ninhydrin and 200 mL of acetic acid. The reaction mixture was boiled in a water bath at 100 °C for 30 min and cooled down at 4 °C for 30 min. Then, 400 mL of toluene was added to the leaf extract and thoroughly mixed. Finally, 120 mL of the toluene phase was removed for an absorbance measurement at 520 nm in a spectrophotometer.

For a soluble sugars analysis, a mixture of 5 mL ethanol 80% (*v*/*v*) and 1.0 g of grounded leaf tissue was prepared and centrifuged at 8000 g for 10 min. Three mL of 0.2% anthrone reagent (0.5 g anthrone in 250 mL of 72% sulfuric acid) was added to 100 mL of ethanolic extract and then incubated in boiling water for 10 min. After cooling into the ice bath, the sample absorbance was read at 620 nm. The total soluble sugars contents were calculated based on a calibration curve and expressed as mg soluble sugars per g fresh weight [48].

### 4.7. Measurements of Relative Electrical Conductivity and TBARS Contents

REC was determined using a portable conductivity meter (HI8733, HANNA, Roma, Italy) by measuring electrolytes leaked from leaves. Sections leaves of the same size were washed three times with distilled water, and were shred into 50 mL tubes containing distilled water. The initial conductivity was recorded as R1. The total conductivity was determined after boiling leaves in 100 °C for 30 min and was recorded as R2. REC was calculated using the following formula: REC (%) = (R1/R2) × 100% [49].

Lipid peroxidation was determined as TBARS contents according to the method of Heath and Packer with minor modifications by using a microplate spectrophotometer (Epoch2, BioTek, Winooski, VT, USA). The absorbance of the supernatant was measured at 535 nm and corrected by subtracting the absorbance at 600 nm. The TBARS contents were calculated by using the extinction coefficient of 155 mM^−1^ cm^−1^) [50].

### 4.8. Statistical Analysis

All data were subjected to analysis of variance (ANOVA) procedures using SPSS Statistics 26 (26, IBM, New York, NY, USA). Plots and tables were drawn using GraphPad Prism 9 (GraphPad Software, Boston, MA, USA). Mean and SE values represent the five biological replicates of each treatment. Duncan’s multiple comparison tests analyzed the difference among means. *p* < 0.05 were considered significant.

## 5. Conclusions

As a shade plant, *Hv* generally exhibited the lower photosynthetic capacity with a higher Pn than the sun plant *Lp*. Furthermore, *Hv* preferred to use the absorbed light energy for carbon assimilation by maintaining PSII and PSI photochemistry, while *Lp* would like to sharply decline the energy utilization to photosynthesis and consume the excess energy by thermal dissipation and cyclic electron flow during CA. As a result, there were serious ROS bursts and lipid peroxidation in *Lp* after CA and the FTR treatments, indicating a much lower freezing tolerance in *Lp* than *Hv*. Additionally, more soluble sugars were accumulated in *Hv* than *Lp*, which contributed more osmoregulatory substances to improve the freezing tolerance in *Hv*. Our study highlights the different photosynthetic strategies and osmoregulator accumulation between sun and shade plants during CA, which eventually led to the different freezing tolerance after the FTR. These findings will be beneficial as they decipher the divergent photoprotective mechanism between the sun and shade plants in response to low temperature.

## Figures and Tables

**Table 1:** The leaf light and CO_2_ response parameters of *Lupinus polyphyllus* and *Helleborus viridis* after 14 d of cold acclimation. AQE: apparent quantum yields, LSP: light saturation point, LCP: light compensation point, Rd: dark respiration rate, CE: the values of carboxylation efficiency, CSP: CO_2_ saturation point, CCP: CO_2_ compensation point, and R_p_: photorespiration rate. Values are the means of five biological replicates ± SE (*n* = 5). Means followed by the different letters indicate the significant differences among the treatment at *p* < 0.05.

Parameter	NA-*Lp*	NA-*Hv*	CA-*Lp*	CA-*Hv*
AQE	0.085 ± 0.004 a	0.070 ± 0.007 a	0.035 ± 0.006 b	0.042 ± 0.005 b
LSP	1315 ± 59 a	804 ± 41 b	865 ± 69 b	690 ± 21 c
LCP	76.4 ± 8.8 a	66.6 ± 0.8 b	36.0 ± 1.7 c	46.8 ± 1.7 c
Rd	2.36 ± 0.30 a	2.47 ± 0.33 a	1.46 ± 0.30 b	2.16 ± 0.30 a
CE	0.128 ± 0.009 a	0.106 ± 0.003 b	0.066 ± 0.005 c	0.070 ± 0.003 c
CSP	1189 ± 40 a	1007 ± 33 b	1070 ± 50 ab	1091 ± 22 ab
CCP	76.1 ± 4.8 b	57.6 ± 5.4 c	98.9 ± 2.5 a	90.3 ± 3.9 ab
R_p_	5.18 ± 0.46 a	3.92 ± 1.41 a	1.38 ± 0.54 b	4.23 ± 0.31 a

## Data Availability

All data are included in the main text.

## References

[1] Boyer J.S. (1982). Plant productivity and environment. Science.

[2] Sanchez B., Rasmussen A., Porter J.R. (2014). Temperatures and the growth and development of *maize* and *rice*: A review. Glob. Chang. Biol..

[3] Yang H., Qiao K.W., Teng J.J., Chen J.B., Zhong Y.L., Rao L.Q., Xiong X.Y., Li H. (2023). Protease Inhibitor ASP Enhances Freezing Tolerance by Inhibiting Protein Degradation in *Kumquat*. Hortic. Res..

[4] Aslam M., Fakher B., Ashraf M.A., Cheng Y., Wang B., Qin Y. (2022). Plant Low-Temperature Stress: Signaling and Response. Agronomy.

[5] Chang C.Y.Y., Brautigam K. (2021). Champions of winter survival: Cold acclimation and molecular regulation of cold hardiness in evergreen conifers. New Phytol..

[6] Ding Y., Shi Y., Yang S. (2019). Advances and challenges in uncovering cold tolerance regulatory mechanisms in plants. New Phytol..

[7] Liu B., Zhao F.M., Cao Y., Wang X.Y., Li Z., Shentu Y., Zhou H., Xia Y.P. (2022). Photoprotection contributes to freezing tolerance as revealed by RNA-seq profiling of rhododendron leaves during cold acclimation and deacclimation over time. Hortic. Res..

[8] Ensminger I., Busch F., Huner N.P.A. (2006). Photostasis and cold acclimation: Sensing low temperature through photosynthesis. Physiol. Plant..

[9] Didaran F., Kordrostami M., Ghasemi-Soltani A.A., Pashkovskiy P., Kreslavski V., Kuznetsov V., Allakhverdiev S.I. (2024). The mechanisms of photoinhibition and repair in plants under high light conditions and interplay with abiotic stressors. J. Photochem. Photobiol. B Biol..

[10] Wei Y., Chen H., Wang L., Zhao Q., Wang D., Zhang T. (2022). Cold acclimation alleviates cold stress-induced PSII inhibition and oxidative damage in tobacco leaves. Plant Signal. Behav..

[11] Ivanov A., Sane P., Zeinalov Y., Simidjiev I., Huner N., Öquist G. (2002). Seasonal responses of photosynthetic electron transport in Scots pine (*Pinus sylvestris* L.) studied by thermoluminescence. Planta.

[12] Sonoike K. (2011). Photoinhibition of photosystem I. Physiol. Plant..

[13] Kozuleva M. (2022). Recent advances in the understanding of superoxide anion radical formation in the photosynthetic electron transport chain. Acta Physiol. Plant..

[14] Gill S.S., Tuteja N. (2010). Reactive oxygen species and antioxidant machinery in abiotic stress tolerance in crop plants. Plant Physiol. Biochem..

[15] Xu C., Wang Y., Yang H., Tang Y., Liu B., Hu X., Hu Z. (2023). Cold acclimation alleviates photosynthetic inhibition and oxidative damage induced by cold stress in *citrus* seedlings. Plant Signal. Behav..

[16] Poorter H., Niinemets Ü., Ntagkas N., Siebenkäs A., Mäenpää M., Matsubara S., Pons T. (2019). A meta-analysis of plant responses to light intensity for 70 traits ranging from molecules to whole plant performance. New Phytol..

[17] Valladares F., Niinemets Ü. (2008). Shade Tolerance, a Key Plant Feature of Complex Nature and Consequences. Annu. Rev. Ecol. Evol. Syst..

[18] Mathur S., Jain L., Jajoo A. (2018). Photosynthetic efficiency in sun and shade plants. Photosynthetica.

[19] Huang D., Wu L., Chen J.R., Dong L. (2011). Morphological plasticity, photosynthesis and chlorophyll fluorescence of *Athyrium pachyphlebium* at different shade levels. Photosynthetica.

[20] Matsubara S., Krause G.H., Aranda J., Virgo A., Beisel K.G., Jahns P., Winter K. (2009). Sun-shade patterns of leaf carotenoid composition in 86 species of neotropical forest plants. Funct. Plant Biol..

[21] Allen D.J., Ort D.R. (2001). Impacts of chilling temperatures on photosynthesis in warm-climate plants. Trends Plant Sci..

[22] Farquhar G.D., Sharkey T.D. (1982). Stomatal Conductance and Photosynthesis. Annu. Rev. Plant Biol..

[23] Genty B., Briantais J.M., Baker N.R. (1989). The relationship between the quantum yield of photosynthetic electron transport and quenching of chlorophyll fluorescence. Biochim. Biophys. Acta.

[24] Yang Y.J., Chang W., Huang W., Zhang S.B., Hu H. (2017). The effects of chilling-light stress on photosystems I and II in three *Paphiopedilum* species. Bot. Stud..

[25] Müller P., Li X.P., Niyogi K.K. (2001). Non-Photochemical Quenching. A Response to Excess Light Energy. Plant Physiol..

[26] Habibi G. (2019). Effects of High Light and Chilling Stress on Photosystem II Efficiency of Aloe Vera L. Plants Probing by Chlorophyll a Fluorescence Measurements. Iran. J. Sci. Technol. Trans. Sci..

[27] Shi S., Shi R., Zhou D., Li T., De K., Gao X., Ma J., Wang F. (2023). Reduction of PSII photosynthetic performance by low temperature is the reason for the growing inhibition of *Kobresia pygmaea* Clarke. Braz. J. Bot..

[28] Gray G.R., Chauvin L.P., Sarhan F., Huner N.P.A. (1997). Cold acclimation and freezing tolerance—A complex interaction of light and temperature. Plant Physiol..

[29] Savitch L.V., Ivanov A.G., Gudynaite-Savitch L., Huner N.P., Simmonds J. (2011). Cold stress effects on PSI photochemistry in *Zea mays*: Differential increase of FQR-dependent cyclic electron flow and functional implications. Plant Cell Physiol..

[30] Rumeau D., Peltier G., Cournac L. (2007). Chlororespiration and cyclic electron flow around PSI during photosynthesis and plant stress response. Plant Cell Environ..

[31] Krämer M., Blanco N.E., Penzler J.F., Davis G.A., Brandt B., Leister D., Kunz H.H. (2024). Cyclic electron flow compensates loss of *PGDH3* and concomitant stromal NADH reduction. Sci. Rep..

[32] Lu J.Y., Nawaz M.A., Wei N.N., Cheng F., Bie Z.L. (2020). Suboptimal Temperature Acclimation Enhances Chilling Tolerance by Improving Photosynthetic Adaptability and Osmoregulation Ability in Watermelon. Hortic. Plant J..

[33] Lu J., Wang Z., Yang X., Wang F., Qi M., Li T., Liu Y. (2020). Cyclic electron flow protects photosystem I donor side under low night temperature in tomato. Environ. Exp. Bot..

[34] Shankar P.S., Parida P., Bhardwaj R., Yadav A., Swapnil P., Seth C.S., Meena M. (2024). Deciphering molecular regulation of reactive oxygen species (ROS) and reactive nitrogen species (RNS) signaling networks in *Oryza* genus amid environmental stress. Plant Cell Rep..

[35] Guo Z., Cai L., Liu C., Chen Z., Guan S., Ma W., Pan G. (2022). Low-temperature stress affects reactive oxygen species, osmotic adjustment substances, and antioxidants in rice (*Oryza sativa* L.) at the reproductive stage. Sci. Rep..

[36] Pinnola A., Bassi R. (2018). Molecular mechanisms involved in plant photoprotection. Biochem. Soc. Trans..

[37] Feng Y., Zhi L., Pan H., Chen Y., Xu J. (2023). Shade Improves Seedling Quality of Ornamental *Cyclocarya* Species under Plastic Greenhouse Cultivation. Ornam. Plant Res..

[38] Cao J., Bao J., Lan S., Qin X., Ma S., Li S. (2024). Research Progress on Low-Temperature Stress Response Mechanisms and Mitigation Strategies in Plants. Plant Growth Regul..

[39] Hornyák M., Grzesiak M., Płażek A., Betekhtin A., Pinski A. (2024). Measurements of Leaf Gas-Exchange Parameters Using Portable CIRAS-3 Infrared Gas Analyzer, with a Parkinson Leaf Chamber (PLC6). Buckwheat.

[40] Yin C.Y., Berninger F., Li C.Y. (2006). Photosynthetic responses of *Populus przewalski* subjected to drought stress. Photosynthetica.

[41] Jian Z.Y., Zhou X.R., Tian J. (2020). Photosynthetic and Chlorophyll Fluorescence Characteristics of *Isodon rubescens* (Hemsley) H. Hara. Sci. Rep..

[42] Ye Z.P. (2007). Application of light-response model in estimating the photosynthesis of super-hybrid rice combination-II Youming 86. Chin. J. Ecol..

[43] Maxwell K., Johnson G.N. (2000). Chlorophyll fluorescence-a practical guide. J. Exp. Bot..

[44] Liu L., Liu Y., Cui J., Liu H., Liu Y.B., Qiao W.L., Sun H., Yan C.D. (2013). Oxidative stress induces gastric submucosal arteriolar dysfunction in the elderly. World J. Gastroenterol..

[45] Guo X., Yu X., Xu Z., Liu L., Liu Y., Qiao W., Sun H., Yan C. (2022). A CC-type glutaredoxin, *MeGRXC3*, associates with catalases and negatively regulates drought tolerance in cassava (*Manihot esculenta* Crantz). Plant Biotechnol. J..

[46] Yang F.W., Zhang H.B., Wang Y., He G., Wang J., Guo D., Li T., Sun G., Zhang H. (2021). The role of antioxidant mechanism in photosynthesis under heavy metals Cd or Zn exposure in tobacco leaves. J. Plant Interact..

[47] Bates L.S., Waldren R.P., Teare I.D. (1973). Rapid determination of free proline for water-stress studies. Plant Soil.

[48] Gilmour S.J., Sebolt A.M. (2000). Overexpression of the *Arabidopsis CBF3* transcriptional activator mimics multiple biochemical changes associated with cold acclimation. Plant Physiol..

[49] Li G.W., Zhang M.H., Cai W.M., Sun W.N., Su W.A. (2008). Characterization of *OsPIP2;7*, a Water Channel Protein in Rice. Plant Cell Physiol..

[50] Heath R.L., Packer L. (1968). Photoperoxidation in isolated chloroplasts. I. Kinetics and stoichiometry of fatty acid peroxidation. Arch. Biochem. Biophys..

